# Identification of featured necroptosis-related genes and imbalanced immune infiltration in sepsis *via* machine learning

**DOI:** 10.3389/fgene.2023.1158029

**Published:** 2023-04-06

**Authors:** Han She, Lei Tan, Ruibo Yang, Jie Zheng, Yi Wang, Yuanlin Du, Xiaoyong Peng, Qinghui Li, Haibin Lu, Xinming Xiang, Yi Hu, Liangming Liu, Tao Li

**Affiliations:** ^1^ State Key Laboratory of Trauma, Burns and Combined Injury, Shock and Transfusion Department, Daping Hospital, Army Medical University, Chongqing, China; ^2^ Department of Anesthesiology, Daping Hospital, Army Medical University, Chongqing, China; ^3^ School of Medicine, Chongqing University, Chongqing, China; ^4^ Department of Intensive Care Unit, Daping Hospital, Army Medical University, Chongqing, China

**Keywords:** sepsis, necroptosis, machine learning algorithm, immune cell infiltration, nomogram

## Abstract

**Background:** The precise diagnostic and prognostic biological markers were needed in immunotherapy for sepsis. Considering the role of necroptosis and immune cell infiltration in sepsis, differentially expressed necroptosis-related genes (DE-NRGs) were identified, and the relationship between DE-NRGs and the immune microenvironment in sepsis was analyzed.

**Methods:** Machine learning algorithms were applied for screening hub genes related to necroptosis in the training cohort. CIBERSORT algorithms were employed for immune infiltration landscape analysis. Then, the diagnostic value of these hub genes was verified by the receiver operating characteristic (ROC) curve and nomogram. In addition, consensus clustering was applied to divide the septic patients into different subgroups, and quantitative real-time PCR was used to detect the mRNA levels of the hub genes between septic patients (SP) (*n* = 30) and healthy controls (HC) (*n* = 15). Finally, a multivariate prediction model based on heart rate, temperature, white blood count and 4 hub genes was established.

**Results:** A total of 47 DE-NRGs were identified between SP and HC and 4 hub genes (*BACH2*, *GATA3*, *LEF1*, and *BCL2*) relevant to necroptosis were screened out *via* multiple machine learning algorithms. The high diagnostic value of these hub genes was validated by the ROC curve and Nomogram model. Besides, the immune scores, correlation analysis and immune cell infiltrations suggested an immunosuppressive microenvironment in sepsis. Septic patients were divided into 2 clusters based on the expressions of hub genes using consensus clustering, and the immune microenvironment landscapes and immune function between the 2 clusters were significantly different. The mRNA levels of the 4 hub genes significantly decreased in SP as compared with HC. The area under the curve (AUC) was better in the multivariate prediction model than in other indicators.

**Conclusion:** This study indicated that these necroptosis hub genes might have great potential in prognosis prediction and personalized immunotherapy for sepsis.

## Introduction

Sepsis is defined as a life-threatening multiple-organ dysfunction caused by the overwhelming response to infection (Galley et al., 2022). It’s estimated that 31.5 million individuals suffer from sepsis annually worldwide, and 5.3 million die from it (Rudd et al., 2020). As a major public health concern, hospitalization expenses from sepsis have been the most expensive in America for several years, which accounted for more than $38 billion of total US hospital costs in 2017 (van den Berg et al., 2022). Despite significant strides in the diagnosis and treatment of sepsis, its incidence continues to rise, underscoring the critical need for a deeper understanding of its pathogenesis. (Li L. et al., 2022). More sensitive and specific targets for diagnosis and therapy must be identified through continued research efforts, as the complexities of sepsis demand a comprehensive approach to ensure effective management and prevention.

Necrosis, the uncontrolled death of cells, is commonly characterized by cell and organelle swelling, loss of plasma membrane integrity, and the release of cell contents, leading to inflammation and tissue damage (Luo et al., 2022; Preston et al., 2022). However, recent studies have highlighted receptor-interacting protein (RIP) kinase-mediated necrosis as a novel form of regulatory necrosis, known as necroptosis (Peng et al., 2022). Necroptosis involves the activation of RIP1, along with two downstream mediators, RIPK3 and mixed lineage kinase domain like (MLKL), and can be inhibited by the pre-apoptotic caspase-8 (Liu et al., 2021; Al-Botaty et al., 2022). Recently, it has been reported that necroptosis is related to the pathogenesis of many diseases, including sepsis (Maremonti et al., 2022). Moreover, increasing studies suggested that inhibition of necroptosis might be a promising target in treating sepsis. However, the role of necroptosis-related genes (NRGs) in sepsis is still unclear.

The dysregulation of the immune system is a pivotal mechanism underlying sepsis. During the initial phase of sepsis, the exaggerated inflammatory response triggers the recruitment of a large number of neutrophils, which play a crucial role in pathogenic bacterial clearance but also cause tissue damage (Ioannou et al., 2022). The damaged cells can promote the inflammatory response as endogenous inflammatory inducers. In addition, M1 macrophages also release excessive proinflammatory cytokines, such as IL-1, IL-8, TNF-α, and IFN-γ (Gharamti et al., 2022). Furthermore, dendritic cells (DCs) can activate toll-like receptors (TLRs) and generate excessive pro-inflammatory factors, which augment the immune response. These over-activated responses ultimately lead to the formation of an “inflammatory storm,” which significantly impairs normal immune function (Xiao et al., 2018). With the development of sepsis, the over activation stage gradually transforms into the immune paralysis or immunosuppression stage (Zhang C. Y. et al., 2019; Forceville et al., 2021). This stage is characterized by increased apoptosis of neutrophils and T cells, immature DCs secreting IL-10 to inhibit immune responses, differentiation of regulatory T cells that secrete IL-10, IL-35, and TGF-β, and an evident imbalance in helper T cell subsets. These work together to make the body lose its normal immune function. Therefore, it is of great significance to improve the immune microenvironment for the treatment of septic patients, and immunotherapy has a broad prospect for clinical application in sepsis. But in sepsis, the relationship between immune infiltration characterization and necroptosis remains unknown.

Machine learning is one of the most important branches of artificial intelligence. By automatically learning the internal pattern of data by feature extraction, machine learning can constantly improve its performance (Chen S. et al., 2022; Monti et al., 2022). The main algorithms include random forest (RF), decision tree (DT), artificial neural network (ANN), and support vector machine (SVM) (Mukherjee et al., 2022; Zaitsev et al., 2022; Zhu et al., 2022; Amiri et al., 2023). Rather than the traditional diagnosis and treatment mode, machine learning provides a totally new insight to improve diagnosis efficiency and more objective and personalized evaluation for patients (Du et al., 2022; Song and Zhao, 2022). Although machine learning has become particularly prominent in the field of diagnosis, it is rarely used in identifying potential diagnostic and prognostic targets in sepsis.

We aimed to identify novel necroptosis-related hub genes in sepsis *via* machine learning algorithms. The role of these NRGs in immune cell infiltration features was investigated to gain more information on the underlying molecular immune mechanisms during the development of sepsis and to discover the possible intervention treatment for sepsis.

## Materials and methods

### Dataset collection

In this study, three datasets included GSE65682, GSE95233, and GSE54514, which were downloaded from the Gene Expression Omnibus (GEO) database (https://www.ncbi.nlm.nih.gov/geo/) were collected for the subsequent analysis. The GSE65682 which contains 760 septic patients and 42 healthy controls was used as the training cohort for screening diagnostic necroptosis-associated genes and subsequent immune infiltration and correlation analysis. Additionally, GSE95233 (included 51 septic patients and 22 healthy volunteers) and GSE54514 (included 35 septic patients and 18 healthy volunteers) were merged as the validation cohort to verify the mRNA expression and the diagnostic performances of hub genes. R packages “SVA” was applied to normalize and remove the batch effects of these gene matrices (Chen Z. et al., 2022). According to the dataset probe annotation files, all these dataset probes were transformed into corresponding gene symbols using Perl scripts.

### Population recruitment

The human study was approved by the Ethics Committee of the Research Institute of Surgery and was registered by the Chinese Clinical Trial Registry (ChiCTR2200055772). A total of 30 septic patients, who were diagnosed by the Sepsis-3 criteria reference for sepsis and septic shock, were recruited from Daping Hospital, Army Medical University (The inclusion and exclusion criteria of septic patients were shown in the Supplementary Table S1.) Fifteen age-matched healthy volunteers were enrolled from the State Key Laboratory of Trauma, Burns, and Combined Injury. All the participants were admitted from December 2021 to April 2022 and provided written informed consent prior to inclusion in this study. A 10 mL volume of blood samples from septic patients were collected within 24 h after admission, and the samples of healthy controls were collected on the enrollment.

### Identification of differentially expressed necroptosis-related genes (DE-NRGs)

A total of 67 necroptosis-related genes (NRGs) were obtained from the Molecular Signatures Database (MSigDB database) (https://www.gsea-msigdb.org/gsea/msigdb/genesets.jsp?collection=H) using the keyword “necroptosis” for further investigation (Supplementary Table S2). Next, the expression matrix of the training cohort (GSE65682) was extracted using the R package “GEOquery”. Then, the “limma” package in the R statistical package was conducted for the analysis of DE-NRGs between septic patients and healthy controls with a threshold set at *p* ≤ 0.05. Finally, these significant DE-NRGs were divided into upregulated genes and downregulated genes by the volcano plot *via* “ggplot2” R packages.

### Functional enrichment analysis of DE-NRGs

The “clusterProfiler” R package was applied to enrich the DE-NRGs into pathways *via* GO and Kyoto Encyclopedia of Genes and Genomes (KEGG) analysis and to verify these enrichment analyses with *p*-value ≤0.05. Then, the biological functions of DE-NRGs were validated by the gene set enrichment analysis using GSEA 4.1.0 software. Significant gene sets were cut off by FDR <0.25 and *p* ≤ 0.05.

### Identification of hub genes *via* LASSO, RF and SVM algorithm

In this study, multiple machine-learning algorithms were applied to search the hub genes. LASSO logistic regression was performed with ten-fold cross-validation to screen iteratively reweighted least squares (IRLs) and was performed for 1,000 cycles to select the feature variables based on minimum criteria or 1-se criteria. To avoid over-fitting and achieve reliable accuracy, the random forest (RF) algorithm was performed to select the optimal genes from the training cohort. Genes with an importance score greater than 1 were used for subsequent signature establishment. Through the deletion of support vector machine (SVM) -generated eigenvectors, the optimal variables were screened out for identifying the diagnostic hub genes in sepsis based on the “e1071” package. At last, a Venn diagram was performed to identify the common hub genes.

### Validation of hub genes

The expressions of these hub genes were compared in the training and validation dataset, respectively. Next, the receiver operating characteristic (ROC) curve was employed to further assess the classification performance of hub genes by calculating the area under the ROC curve. A prediction nomogram model was constructed based on these hub genes *via* the R package “rms”, and the prediction capability of the model was validated using ROC analysis. The nomogram score of each sample was calculated according to the following formula in the train data: = (−4.16× the expression of *GATA3*) + (−0.393× the expression of *LEF1*) + (−1.313× the expression of *BACH2*) + (−1.675× the expression of *BCL2*) and (−2.537× the expression of *GATA3*) + (0.006× the expression of *LEF1*) + (−0.396× the expression of *BACH2*) + (0.712× the expression of *BCL2*) in the test data.

### Immune infiltration analysis

To investigate the immune infiltration of healthy controls and sepsis samples, CIBERSORT algorithms were applied based on “CIBERSORT R script v1.03 to determine the proportion of immune cells. The correlation analysis between immune cells and NRGs was performed *via* “ggplot2” R packages. The immune scores were estimated using the R package “estimate”, and the immune function scores were analyzed by the R package “GSVA”.

### Consensus clustering

According to the 4 hub genes, consensus clustering was performed by the R package “ConsensusClusterPlus”. The clustering was established on the grounds of partitioning around medoids with “Euclidean” distances, and the septic patients were clustered into two subtypes according to the optimal classification.

### Quantitative real-time RT-PCR (qRT-PCR)

RNA was extracted from human blood samples by the PureLink™ blood total RNA extraction kit (Invitrogen). The purity and concentration of the extracted RNA were detected using a NanoDrop 2000 ultraviolet-visible spectrophotometer (Thermo). The extracted RNA was then reverse transcribed into cDNA libraries using the Bestar™ qPCR RT Kit (DBI Bioscience), and fluorescent quantitative PCR reactions were performed using the Bestar^®^ SYBRGreen qPCR master mix (DBI Bioscience). The relative RNA expression was calculated with the efficiency corrected 2^−ΔΔCT^ method using *β-actin* as an internal control. Gene-specific primers used in this study were listed in Supplementary Table S3.

### Statistical analysis

All statistical analyses were performed using R software (version 4.1.2). *p* < 0.05 was considered a statistically significant difference. The correlation analysis was adjusted by Pearson’s correlation and their strength was determined by the following absolute value criteria: r = 0.00–0.19 “very weak,” r = 0.20–0.39 “weak,” r = 0.40–0.59 “moderate,” r = 0.60–0.79 “strong,” r = 0.80–1.0 “very strong.”

## Results

### Identification and functional enrichment analysis of DE-NRGs

**FIGURE 1:**
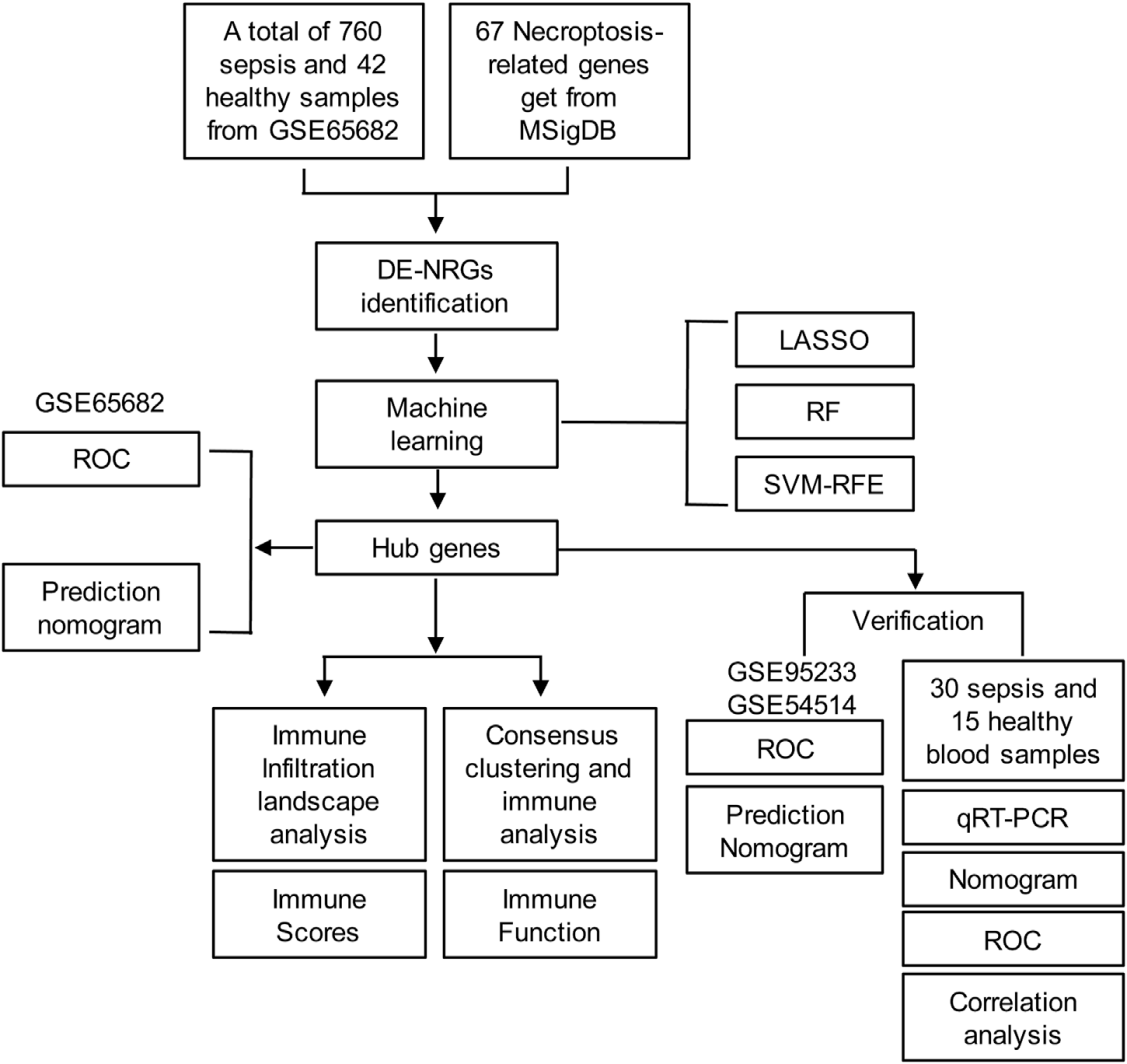
Diagram of the experiment scheme.

**FIGURE 2:**
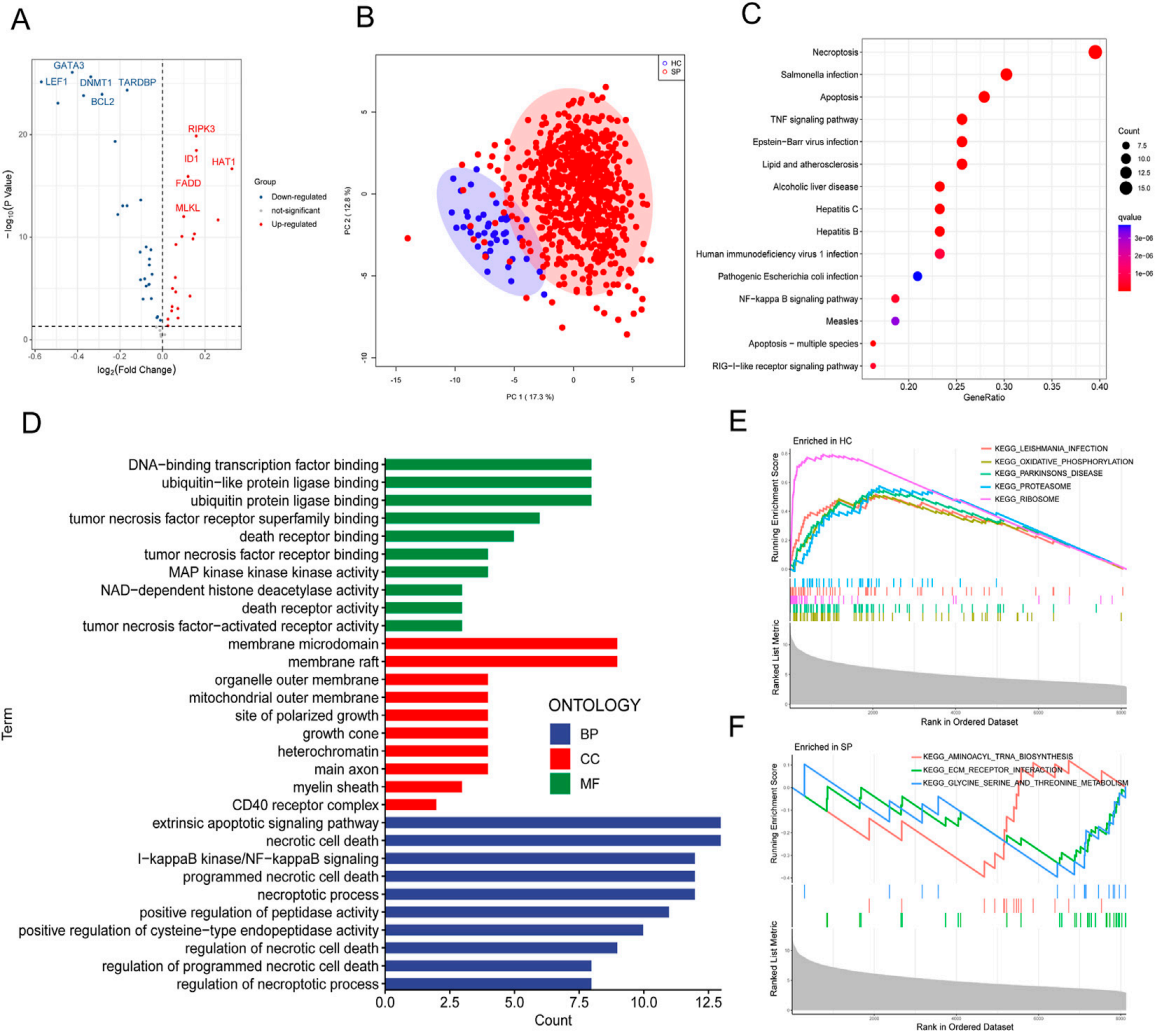
Identification and functional enrichment analysis of DE-NRGs from GEO dataset. **(A)** Volcano plot of DE-NRGs of GSE65682. Blue dots indicated downregulated DE-NRGs while red dots indicate upregulated DE-NRGs. DE-NRGs were identified as those with student’s t-test *p* ≤ 0.05. **(B)** Principal Components Analysis (PCA) score plot of GSE65682. PC1 and PC2 in the figure represent the scores of the first and second principal components respectively. Each scatter represents a sample. The red circle represents septic patients, and the blue circle represents the healthy controls. **(C)** Kyoto Encyclopedia of Genes and Genomes (KEGG) pathway analysis of DE-NRGs. **(D)** Gene ontology (GO) results of biological process (BP) cellular component (CC) and molecular function (MF) of DE-NRGs. Gene set enrichment analysis (GSEA) for DE-NRGs in **(E)** healthy controls and **(F)** septic patients.

The diagram of the experiment scheme was shown in Figure 1. Compared with non-sepsis samples, 67 necroptosis-related genes (NRGs) were involved in GSE65682, of which 20 NRGs were upregulated and 28 were downregulated (*p* ≤ 0.05) (Figure 2A; Supplementary Table S4). Principal component analysis (PCA) illustrated a remarkable separation between the healthy control and sepsis group based on the NRGs (Figure 2B). The results of KEGG pathway analysis showed that the DE-NRGs were mainly enriched in Necroptosis, *Salmonella* infection, Apoptosis, and TNF signaling pathway (Figure 2C). As for GO analysis (Figure 2D), cellular component (CC) showed that DE-NRGs were mainly distributed in membrane microdomain, membrane raft, organelle outer membrane, site of polarized growth and so on. The top 10 molecular function (MF) included DNA−binding transcription factor binding and ubiquitin−like protein ligase binding, and tumor necrosis factor receptor superfamily binding, etc. The top 10 biological processes (BP) of DE-NRGs were shown in Figure 2D, including extrinsic apoptotic signaling pathway, necrotic cell death, I−kappa B kinase/NF−kappa B signaling, and programmed necrotic cell death events (Figures 2E, F). Additionally, GSEA analysis of DE-NRGs of the healthy controls and septic patients were exhibited in Figures 2E, F, respectively. Patients with sepsis showed over-representation of sub-networks linked to aminoacyl tRNA biosynthesis, and ECM receptor interaction.

### Four common hub genes were screened out *via* machine language algorithm

**FIGURE 3:**
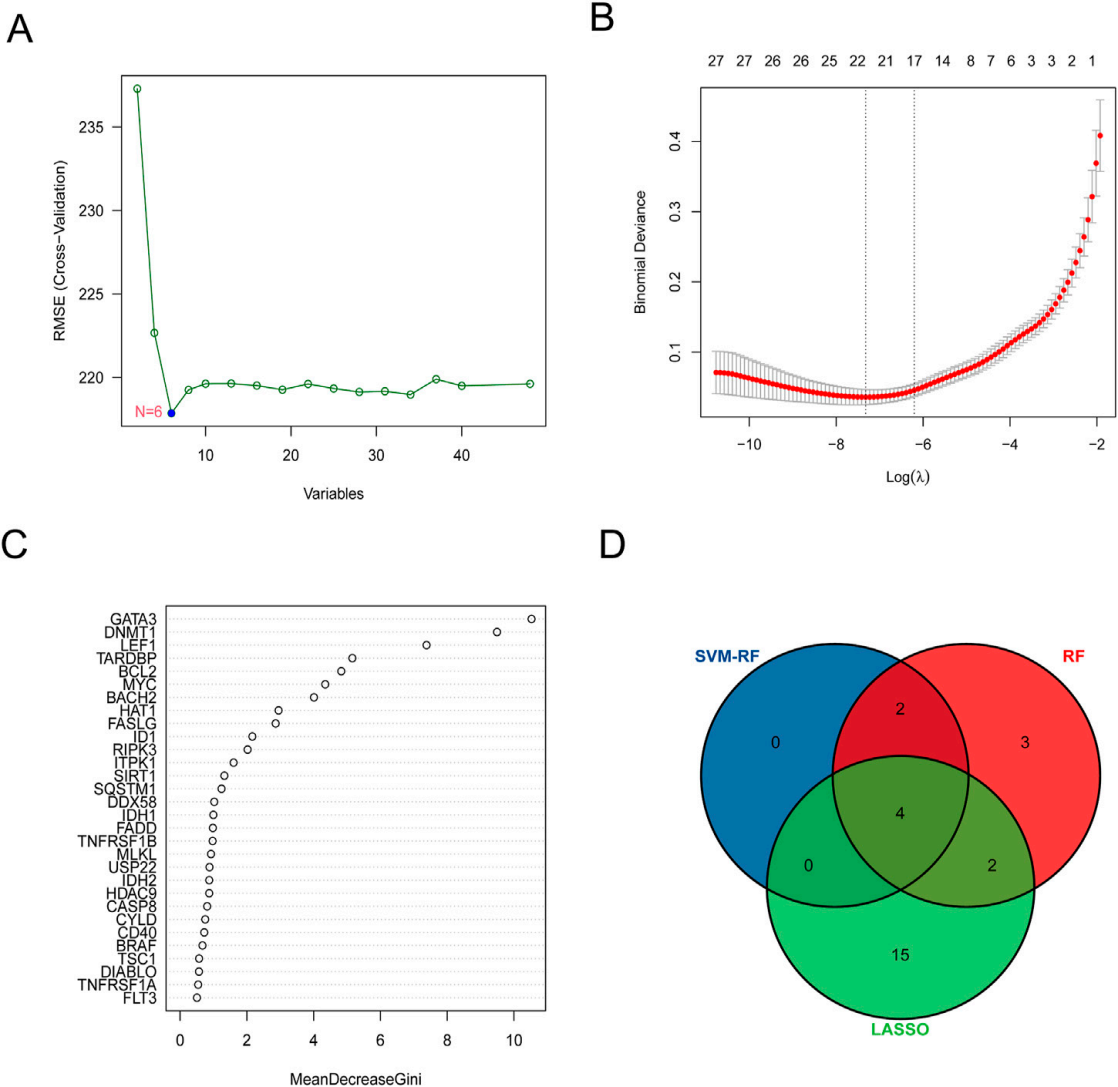
Four common hub genes were screened out *via* machine language algorithm. **(A)** Support vector machine (SVM) was used for screening DE-NRGs. **(B)** Least absolute shrinkage and selection operator (LASSO) logistic regression algorithm to screen DE-NRGs. LASSO logistic regression was performed with 10-fold cross-validation to screen iteratively reweighted least squares (IRLs) and was performed for 1,000 cycles to select the feature variables based on minimum criteria or 1-se criteria. **(C)** Random forest (RF) algorithm was used to screen DE-NRGs. Genes with an importance score greater than 1 were used for subsequent signature establishment. **(D)** VENN diagram of common hub genes.

A total of 6 hub genes were identified by the best support vector *via* the SVM algorithm The best support vector was screened according to the RMSE (root mean square error). The smaller the RMSE, the better the fitting (Figure 3A). LASSO logistic regression was established to shrink the regression coefficients toward zero and select out hub genes. As shown in Figures 3A, B total of 21 hub genes were screened. Likewise, RF was also built for hub genes screening (Figure 3C), and 11 hub genes were screened out. Finally, the intersection of the results of these 3 methods contained the 4 common hub genes (*BACH2, GATA3, LEF1,* and *BCL2*) shown below the Venn diagram (Figure 3D).

### Validation of hub genes in the training and test cohort

**FIGURE 4:**
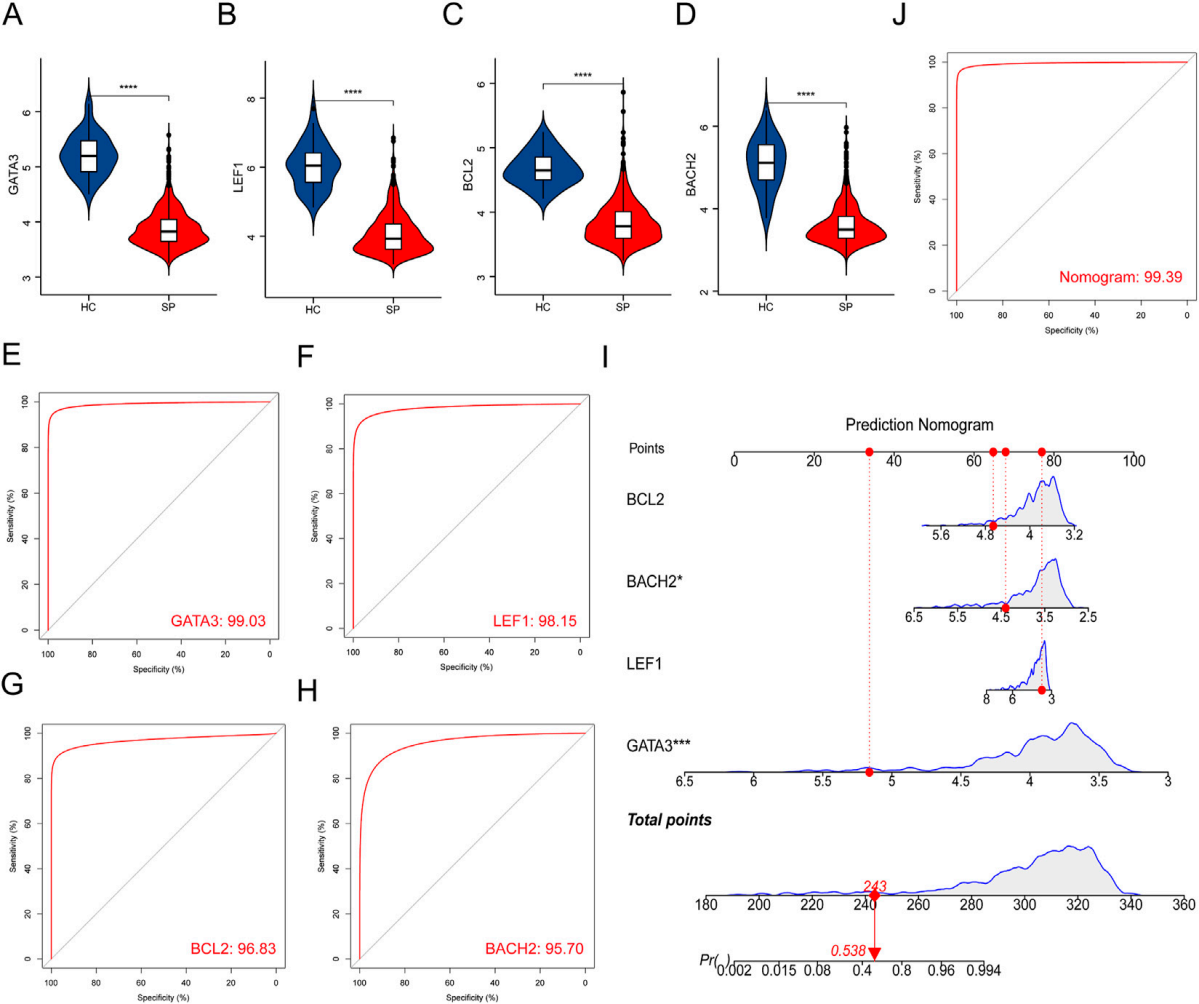
Validation of hub genes in the training cohort. **(A–D)** Validation of the expression of hub genes in patients with sepsis and healthy controls in the training cohort. **(E–H)** ROC curve of hub genes in the training cohort. **(I)** Prediction nomogram model was constructed based on the hub genes in the training cohort. **(J)** ROC curve of the nomogram in the training cohort. ****p < 0.0001.

**FIGURE 5:**
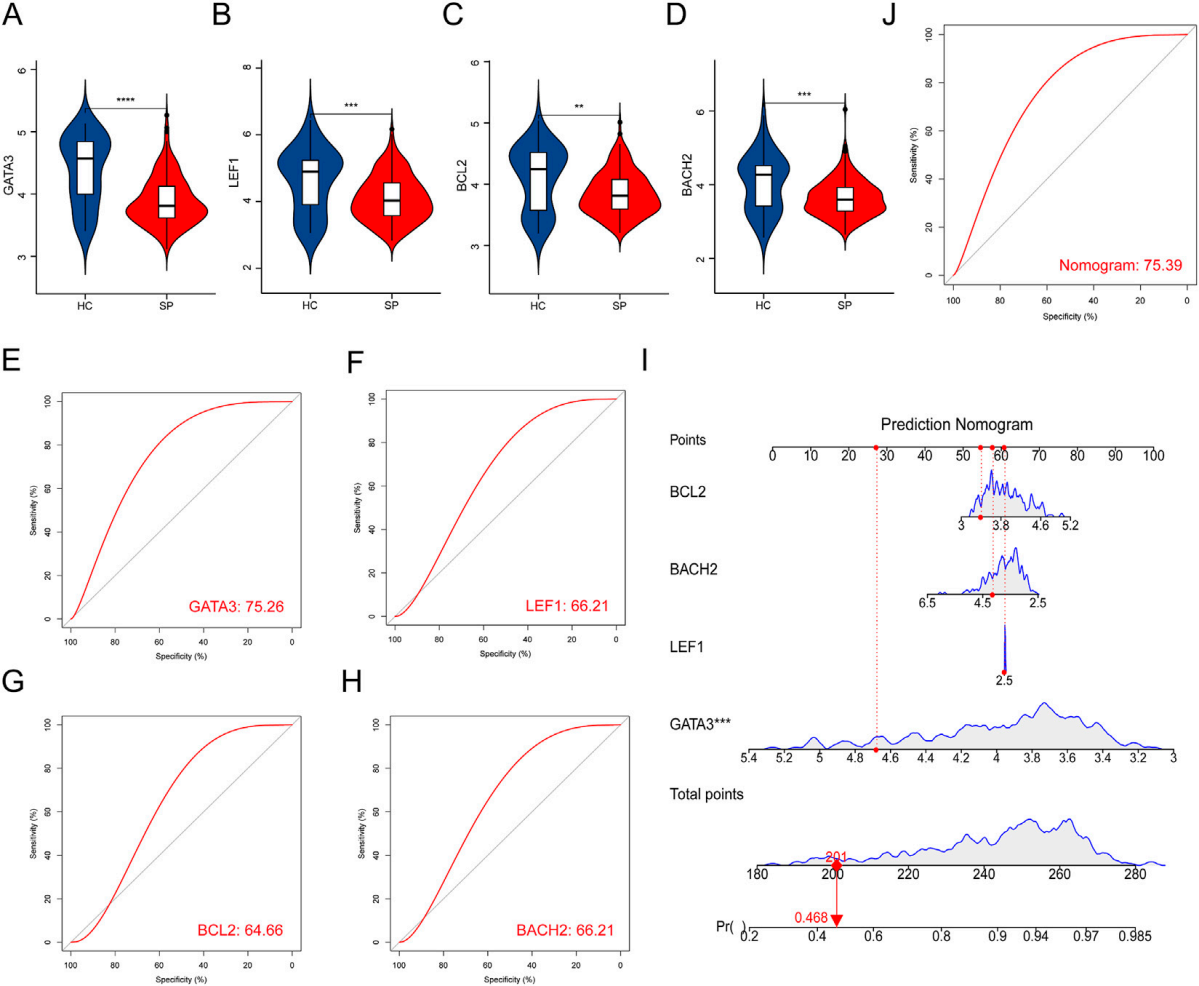
Validation of hub genes in the test cohort. **(A–D)** Validation of the expression of the 4 hub genes in septic patients and healthy controls in the test cohort. **(E–H)** ROC curve of the hub genes in the test cohort. **(I)** Prediction nomogram model was constructed based on the hub genes in the test cohort. **(J)** ROC curve of nomogram in the test cohort. **p < 0.01, ***p < 0.001, ****p < 0.0001.

The expressions of the 4 hub genes were validated in the training cohort (GSE65682) (Figures 4A–D). ROC curves showed that these hub genes had an excellent prediction ability for sepsis with the area under the curve (AUC) ratio>90% (Figures 4E–H). Subsequently, a model of the nomogram was established to evaluate the diagnostic capability of the hub genes (Figure 4I). The ROC of the nomogram was 0.994, which exhibited a satisfactory diagnostic capability of sepsis (Figure 4J). Then, the expressions of these 4 hub genes were validated in the merged test cohort (GSE95233 and GSE54514) (Figures 5A–D). The prediction ability of *BACH2, GATA3, LEF1,* and *BCL2* were validated, with an AUC of 0.662, 0.752, 0.662, and 0.647, respectively (Figures 5E–H). Then, a nomogram model was also established to assess the diagnostic capability of the hub genes (Figure 5I). The ROC of the nomogram was 0.754 (Figure 5J).

### Relationships between the hub genes and infiltrating immune cells

**FIGURE 6:**
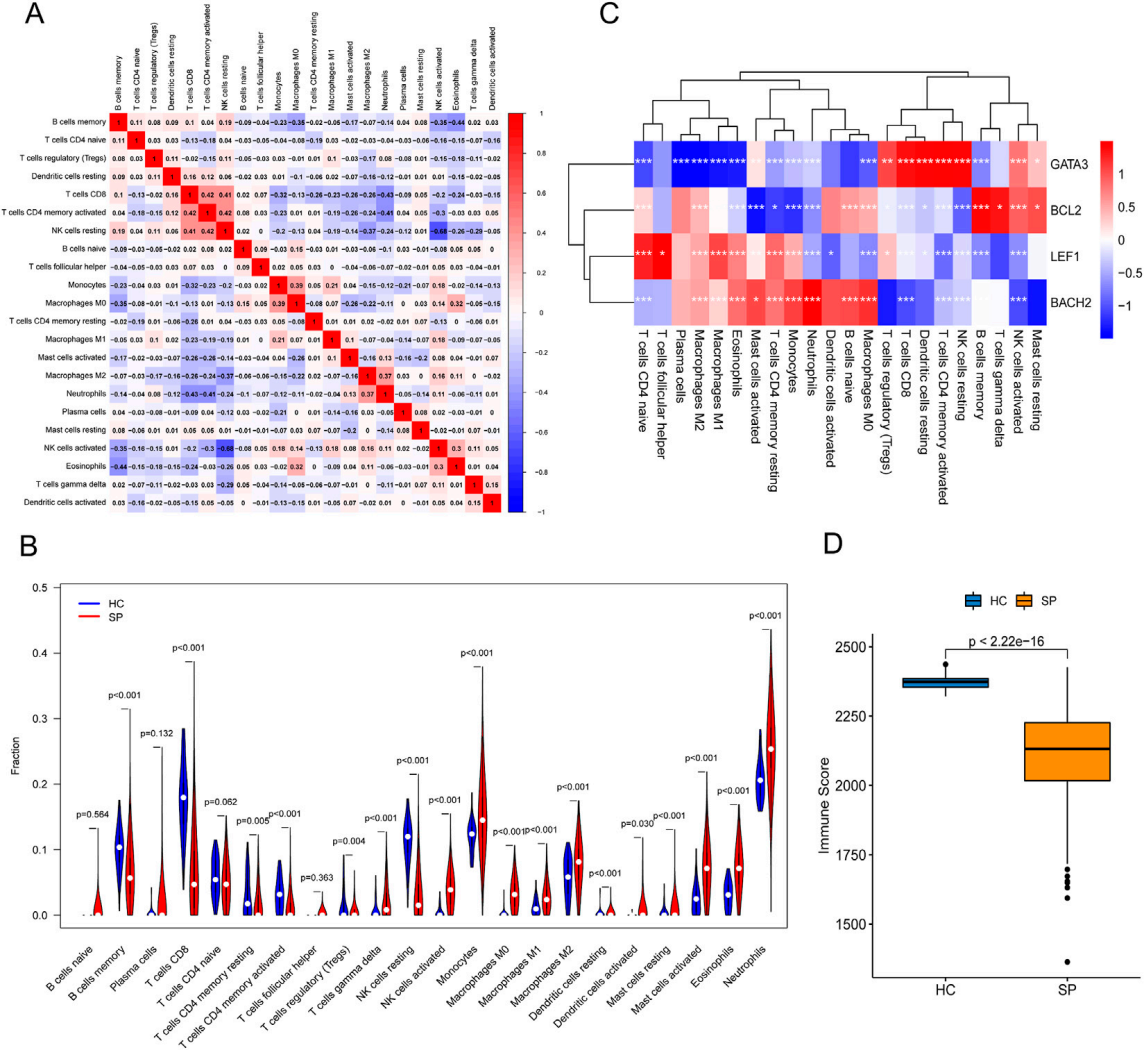
The landscape of Immune cell infiltration and the correlation analysis in training cohort. **(A)** Person’s correlation analysis of 22 types of immune cells. **(B)** Analysis of immune-cell proportion comparisons between septic patients and healthy controls by CIBERSORT. **(C)** Person’s correlation analysis between infiltrating immune cells and the 4 hub genes. Red nodes indicated positive correlation while blue nodes indicated negative correlation. **(D)** Immune scores analyzed by the R package “estimate” between healthy control (HC) and septic patients (SP). *p ≤ 0.05, **p < 0.01, ***p < 0.001.

First, a person’s correlation coefficient analysis of the 22 types of immune cells was shown in the Figure 6A. Then, immune-cell proportion comparisons between septic patients and healthy controls were analyzed in the dataset GSE65682 (Figure 6B). The fractions of memory B cells, CD8 T cells, activated memory CD4 T cells, resting memory CD4 T cells, and Tregs were found to be lower in the septic patients, whereas the fraction of gamma delta T cells, monocytes, activated NK cells, macrophages, resting Mast cells, Eosinophils, and Neutrophils were higher in the healthy controls (Figure 6B). Next, the correlation between immune cells and the 4 hub genes was shown in the correlation thermogram. *GATA3* was found to be positively correlated with the infiltrating Tregs, CD8 T cells, memory activated CD4 T cells, resting NK cells, and resting DCs, while *BCL2* was positively correlated with the memory B cells and activated NK cells. In addition, both *LEF1* and *BACH2* were positively correlated with M1 macrophages, M2 macrophages, monocytes, and Neutrophils (Figure 6C).The immune scores showed that the immune state of immune cells in the SP group was lower than the HC group (Figure 6D). The results of immune scores and correlation between the 4 hub genes and immune-cell infiltrations suggested an immunosuppressive microenvironment in septic patients, and the 4 hub genes were associated with immune infiltration in sepsis.

### Searching for individualized immunotherapy subgroups by consensus clustering

**FIGURE 7:**
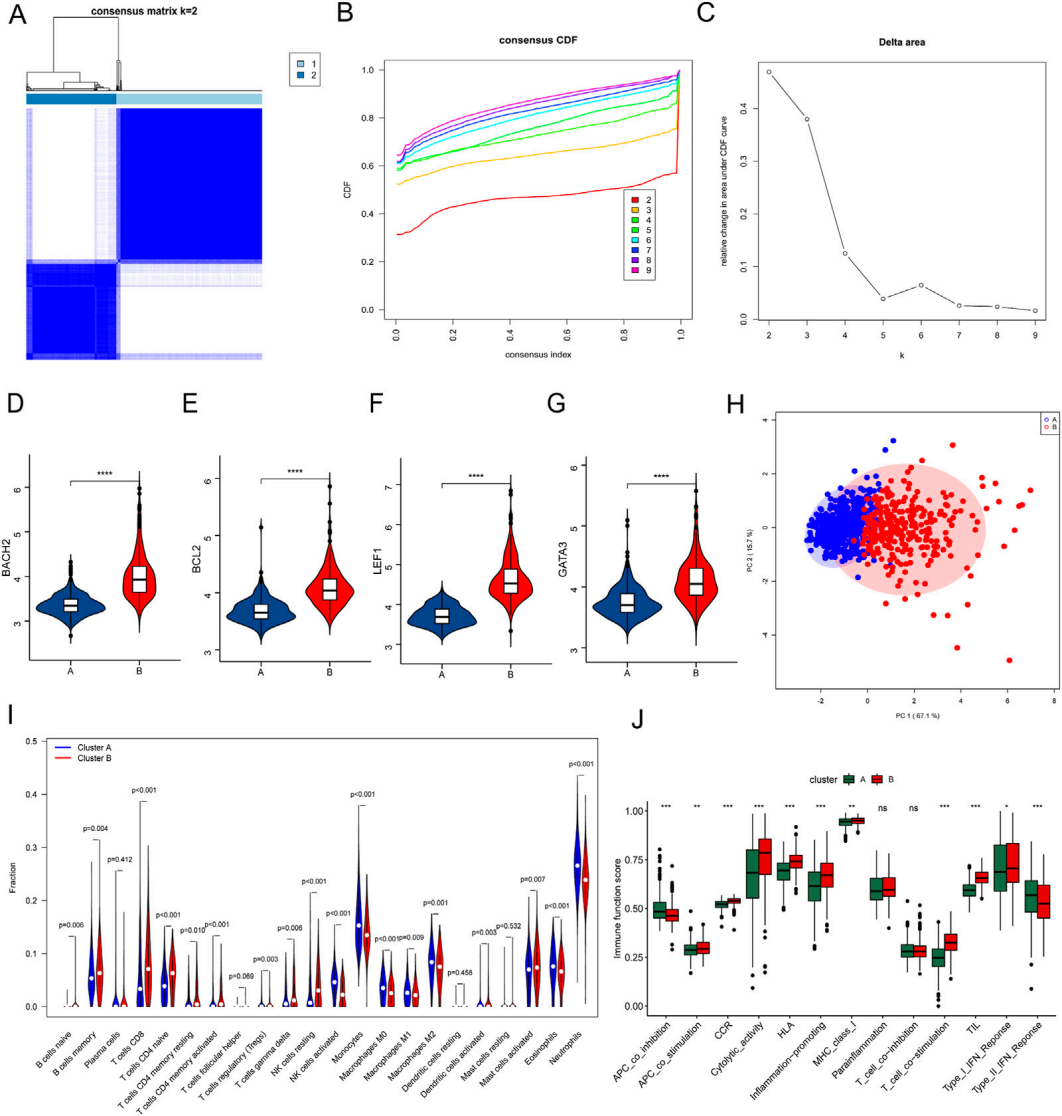
Consensus clustering of septic patients and immune microenvironment landscape analysis. **(A)** Consensus clustering heatmap showed the optimal classification of septic samples with K = 2. **(B)** Consensus CDF. **(C)** Delta area. **(D–G)** Gene expression of the 4 hub genes between Cluster A and Cluster B. **(H)** PCA analysis showed a different distribution pattern in Cluster A and Cluster B. **(I)** The fraction of the 22 types of immune cells in Cluster A and Cluster B. **(J)** The immune function scores between Cluster A and Cluster B. *p ≤ 0.05, **p < 0.01, ***p < 0.001, ****p < 0.0001.

Consensus clustering was performed to cluster the septic patients into 2 subgroups (Figures 7A–C), and there were 470 patients in Custer A, and 290 patients in Cluster B. The expressions of the 4 hub genes were significantly higher in patients in Cluster B than that in Custer A (Figures 7D–G). As shown in Figure 7H, the PCA plot illustrated a remarkable separation between Cluster A and Cluster B. The CIBERSORT results showed that the proportion of Monocytes, Macrophages, Eosinophils, and Neutrophils was higher in patients in Cluster A, whereas the fraction of memory B cells, CD8 T cells, resting memory CD4 T cells, naïve CD4 T cells, gamma delta T cells, resting NK cells, and resting mast cells were higher in patients in Cluster B (Figure 7I). The results of Figure 7J showed that immune functions, APC co-inhibition and Type II IFN response, were lower in the Cluster B than in the Cluster A, while the immune functions such as APC co-stimulation, Cytolytic activity, and T cell co-stimulation were higher in the Cluster B than in the Cluster A (Figure 7J). Based on the difference of immune function between two Clusters, the personalized immunotherapy for septic patients in different subgroups may be provided in the future.

### Integration of hub genes and routine indicators in diagnosis of sepsis

**FIGURE 8:**
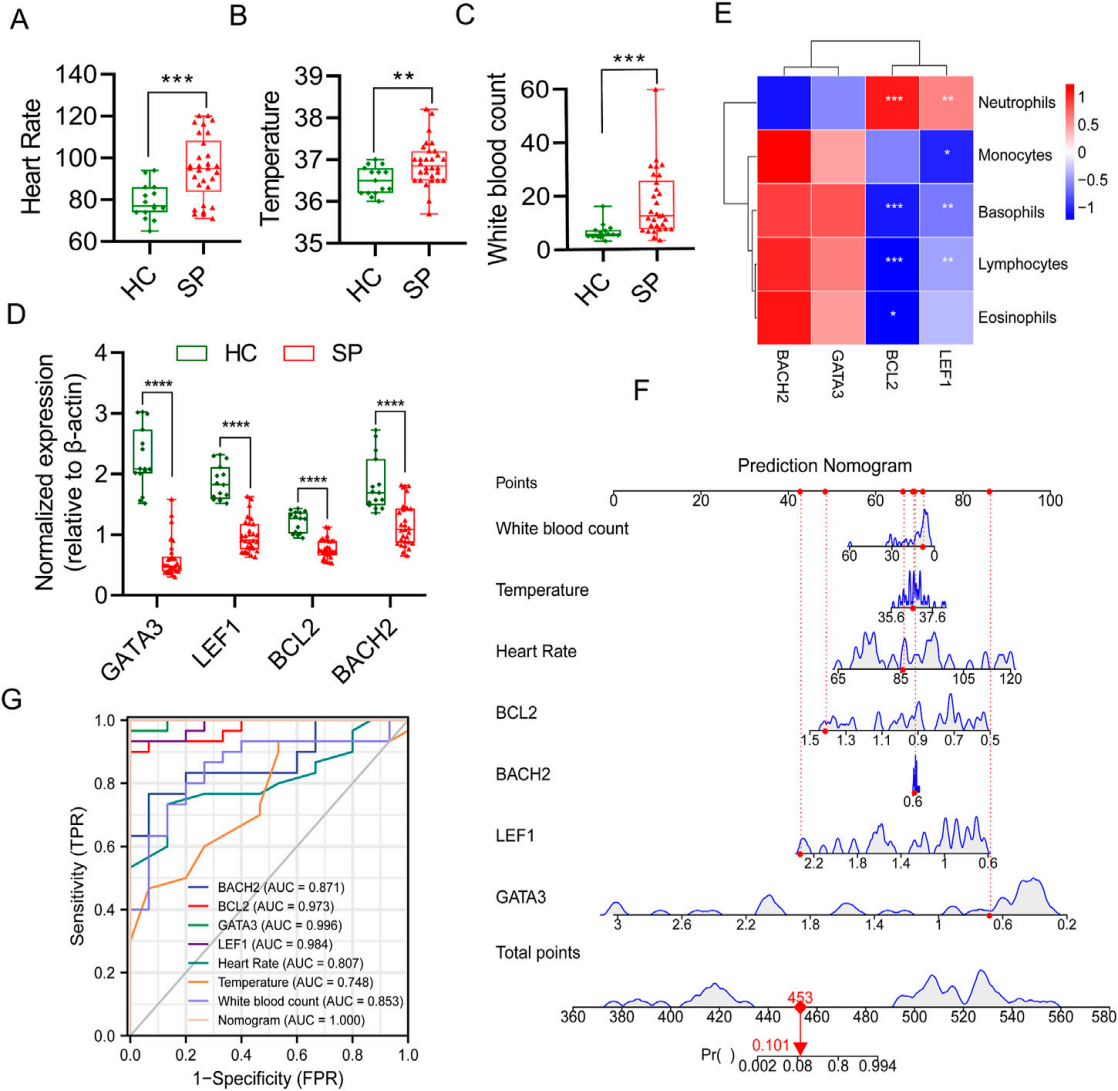
Multivariate prediction nomogram model of sepsis. **(A)** Heart Rate. **(B)** Temperature. **(C)** White blood count. **(D)** Normalized gene expression of the 4 hub genes between HC and SP by qPCR. **(E)** Person’s correlation analysis between immune cells proportion of blood routine and the 4 hub genes. **(F)** Prediction nomogram model was constructed based on multivariate indicators. **(G)** ROC curve of multivariate indicators. *p ≤ 0.05, **p < 0.01, ***p < 0.001, ****p < 0.0001.

To explore the diagnostic value of these hub genes in sepsis, 30 septic patients and 15 healthy controls were recruited (clinical information of septic patients and healthy volunteers was shown in Supplementary Table S5). The 28 days mortality of septic patients was 36.7% (11/30), the ICU mortality was 13.3% (4/30), and another type of mortality was 6.7% (2/30). The heart rate, temperature, and white blood count, which are routine diagnostic indicators of sepsis, were higher in the SP group (Figures 8A–C). The blood routine data between septic patients and healthy controls were collected, and the proportions of lymphocytes and basophils were lower in the SP group than the HC group, while the percent of neutrophils was higher in the SP group (Supplementary Table S6). The results of quantitative real-time PCR showed that the mRNA expression of the four genes was significantly lower in septic patients than those in healthy controls (Figure 8D). The results of Figure 8E showed that the hub genes, *BCL2* and *LEF1*, were positively correlated with neutrophils, and negatively correlated with lymphocytes and basophils. Subsequently, a multivariate prediction nomogram model based on routine diagnostic indicators and 4 hub genes was established (Figure 8F). ROC analysis showed that the AUC of the multivariate prediction nomogram model was highest than other indicators (Figure 8G).

## Discussion

In the present study, we identified 47 DE-NRGs between sepsis and healthy control in the dataset GSE65682 and screened out 4 hub genes (*BACH2, GATA3, LEF1,* and *BCL2*) *via* various machine learning algorithms. By using CIBERSORT and Pearson’s correlation analysis, we found an exhausted microenvironment in sepsis. In addition, septic patients were divided into 2 clusters using consensus analysis, and the difference in immune microenvironment between the 2 clusters provided a theoretical basis for personalized immunotherapy of sepsis. Finally, a multivariate prediction nomogram model based on routine diagnostic indicators and 4 hub genes was established.

As a strictly controlled cell death, necroptosis is mainly regulated by the *RIPK1/RIPK3/MLKL* Pathway, which eventually induces cell death (Afonso et al., 2021). Recent research indicated that the role of necroptosis was closely related to the progress of sepsis, and the prevention of necroptosis could improve the prognosis of sepsis. *RIPK3* could enhance sepsis-induced kidney injury by promoting mitochondrial dysfunction. It’s reported that the inhibition of *RIPK3* or *RIPK1* could reduce systemic inflammation and organ damage in newborn mice with sepsis. A clinical study showed that the plasma level of *HMGB1* was associated with *RIPK3* and *MLKL*, and elevated *HMGB1* ultimately led to poor prognosis in septic patients (Chen et al., 2020; Yoo et al., 2021). Fan et al. found that in the cecal ligation and puncture (CLP) septic mice model, down-regulating the expression of myeloid differentiation factor 2 (MD-2), which was the mediator of crosstalk between apoptosis and necroptosis in neurons, could reduce depressive-like behavior in sepsis-associated encephalopathy (Fan et al., 2022). In the study, we found that 4 necroptosis-related hub genes (*BACH2, GATA3, LEF1,* and *BCL2*) were closely related to sepsis, providing a potential new target for the diagnosis and therapy of sepsis.

*BACH2* is a well-known transcriptional repressor involved in the development and function of innate and adaptive immune cells. For example, *BACH2* is essential during every stage of B cells development, and it could delay class-switch recombination and inhibit the differentiation of plasma cells. *BACH2* could also inhibit the transformation of CD4 T cells into Th2 cells and promote the production of regulatory T (Treg) cells to balance the immune response. It’s reported that *BACH2* controlled the GC program by directly inhibiting the pro-apoptotic protein *BIM*, revealing the precise role of *BACH2* in GC biology (Marroqui et al., 2014; Hu et al., 2022). *BACH2* has been found to be associated with sepsis recently. Elisa et al. found that heme training promoted resistance to sepsis partly by dissociation of *BACH2* (Jentho et al., 2021). In this study, BACH2 was also identified as a hub gene involved in sepsis and associated with immune infiltration.

*GATA3* is the master transcription factor of Th2 lymphocyte differentiation. It can be used as a new target for human obesity immune regulation by reducing macrophage recruitment and inflammation in muscle and liver. Xu et al. found that T-bet and *GATA3* (the specific transcription factor of Th1 and Th2 cells) were significantly downregulated in septic patients and the non-survivors than that in healthy controls and the survivors, respectively, and the sustained low levels of Th1 and Th2 cell subsets suggested the suppression of adaptive immunity, which might be the leading cause of death in septic patients (Xu et al., 2020).

As a member of the T-cell Factor (TCF)/lymphoid enhancer-binding factor 1 (*LEF1*) family of high-mobility group transcription factors, *LEF1* is a mediator of the Wnt/β-catenin signaling pathway but can also regulate gene transcription independently. Zhang et al. found that *LEF1* might involve in the progress of sepsis and sepsis-induced ARDS by analyzing the blood gene expression profiles of septic patients. Therefore, *LEF1*, as a hub gene, may play a crucial role during sepsis and may predict the outcome of septic patients (Zhang J. et al., 2019).

*BCL2* protein contains four highly conserved domains BH 1–4, and functions as an antiapoptotic protein by regulating mitochondrial membrane permeability and cytochrome C release. *BCL2* prevents apoptosis and promotes cellular survival by neutralizing BH3 domain-containing proteins, which can directly activate the pore-forming proteins *BAX* and *BAK*. Besides, *BCL2* can limit the induction of necroptosis by downregulating the *RIP3*-induced phosphorylation of *MLKL* to reduce *MLKL* oligomerization (Campos et al., 2021; Li X. et al., 2022).

Although immunotherapy has made remarkable achievements in tumor therapy over the last decade, the lack of specific therapeutic targets leads to slow progress in immunotherapy for sepsis. Recently, the significant correlation between necroptosis and immune cell infiltration has been proven. In 2016, Aaes et al. confirmed for the first time that necroptosis could be immunogenic (Aaes et al., 2016). They found that the vaccination with necroptotic cancer cells could inhibit tumor growth, and indicated that necroptotic cancer cells could efficiently induce the maturation of dendritic cells, the cross start of cytotoxic T cells, and IFN- γ generation, leading to adaptive immune response. Park et al. identified *TRIM28* as a co-repressor that regulated transcriptional activity during necroptosis (Park et al., 2021). Activated *RIPK3* phosphorylated *TRIM28*, inhibited the chromatin binding activity of *TRIM28*, thus promoting the activation of *NF-κB*. Finally, it led to the increase of cytokine expression to enhance the immune response, such as the maturation of dendritic cells. In the present study, consensus clustering was used to divide the septic patients into two clusters. It was found that the expressions of the hub genes were higher in Cluster B, in which the proportion of B cells, CD8 T cells, CD4 T cells, resting NK cells, and resting mast cells were higher. By contrast, the fractions of Monocytes, Macrophages, and Neutrophils were higher in Cluster A. The difference in immune microenvironment between subgroups of septic patients provides an innovative insight into personalized immunotherapy for sepsis.

## Conclusion

In conclusion, using machine learning analysis we identified 4 necroptosis-related hub genes (*BACH2, GATA3, LEF1,* and *BCL2*), which could be used as the potential di-agnostic and prognostic biological marker in sepsis. Immune infiltration analysis revealed that NRGs might play pivotal roles in immune response during sepsis. Consensus analysis provided a theoretical basis for personalized immunotherapy for sepsis.

## Data Availability

The datasets presented in this study can be found in online repositories. The names of the repository/repositories and accession number(s) can be found in the article/Supplementary Material.
