# Supplementary material for: Identification of featured necroptosis-related genes and imbalanced immune infiltration in sepsis via machine learning
Source: Front Genet. 2023 Apr 6;14:1158029. doi: 10.3389/fgene.2023.1158029 (PMC10117955; doi:10.3389/fgene.2023.1158029)
Supplement: Supplementary file 1 [file Table1.DOCX]

**Supplementary Table 1:** The inclusion and exclusion criteria of septic patients.

| Inclusion criteria | 1. Diagnosed as sepsis. 2. The patient informed consent and participated voluntarily. 3. 18-80 years old (including 80 years old). 4. Register general medical conditions. |
| --- | --- |
| Exclusion criteria | 1. Age>80 years or ≤18 years. 2. Previous history of chronic heart disease, liver and kidney diseases. 3. Pregnant or lactating women. 4. Hyperlipidemia, diabetes, or other metabolic diseases. 5. Complicated with cardiovascular, liver, kidney and hematopoietic system and other serious primary diseases. 6. Combined with psychiatric diseases. 7. Long-term use of sedative drugs or alcohol. 8. Complicated with tumor or immune deficiency and other diseases that have a greater impact on immunity. 9. Patient or family refusal to be involved in the study. |
