# Supplementary material for: Identification of featured necroptosis-related genes and imbalanced immune infiltration in sepsis via machine learning
Source: Front Genet. 2023 Apr 6;14:1158029. doi: 10.3389/fgene.2023.1158029 (PMC10117955; doi:10.3389/fgene.2023.1158029)
Supplement: Supplementary file 2 [file Table2.DOCX]

**Supplementary Table 1:** The gene list of 67 necroptosis-related genes.

|  | **67** | **necroptosis-related** | **genes** |  |
| --- | --- | --- | --- | --- |
| FADD | TRIM11 | TNFSF10 | DNMT1 | PLK1 |
| FAS | CASP8 | TNFRSF1B | CFLAR | MPG |
| FASLG | ZBP1 | TRAF2 | BRAF | BACH2 |
| MLKL | MAPK8 | PANX1 | AXL | GATA3 |
| RIPK1 | IPMK | OTULIN | ID1 | MYCN |
| RIPK3 | ITPK1 | CYLD | CDKN2A | ALK |
| TLR3 | SIRT3 | USP22 | HSPA4 | ATRX |
| TNF | MYC | MAP3K7 | BCL2 | TERT |
| TSC1 | TNFRSF1A | SQSTM1 | STUB1 | SLC39A7 |
| IDH2 | LEF1 | STAT3 | FLT3 | SPATA2 |
| KLF9 | BNIP3 | DIABLO | HAT1 | RNF31 |
| HDAC9 | CD40 | EGFR | SIRT2 | IDH1 |
| HSP90AA1 | BCL2L11 | DDX58 | SIRT1 | TARDBP |
|  |  |  |  | APP |
|  |  |  |  | TNFRSF21 |
