# Supplementary material for: Identification of featured necroptosis-related genes and imbalanced immune infiltration in sepsis via machine learning
Source: Front Genet. 2023 Apr 6;14:1158029. doi: 10.3389/fgene.2023.1158029 (PMC10117955; doi:10.3389/fgene.2023.1158029)
Supplement: Supplementary file 3 [file Table5.DOCX]

**Supplementary Table 5:** Characteristics of the septic patients and healthy controls.

| **Characteristics** |  | **Septic patients** | **Healthy controls** | **p-Value** |
| --- | --- | --- | --- | --- |
|  |  | **(n=30)** | **(n=15)** |  |
| Age, yrs | Mean | 60.1 | 50.4 | 0.053 |
|  | SD | 14.5 | 17.2 |  |
| Gender, n (%) | Female | 10 (33.3) | 6 (40.0) | 0.66 |
|  | Male | 20 (66.7) | 9 (60.0) |  |
| Temperature, ℃ | Mean | 36.9 | 36.5 | 0.01 |
|  | SD | 0.5 | 0.3 |  |
| Heart rate/min | Mean | 94.9 | 79.1 | 0.001 |
|  | SD | 15.3 | 8.3 |  |
| White blood cell count, ×10^9^/L | Mean | 16.9 | 6.6 | 0.003 |
|  | SD | 12.2 | 3.1 |  |
| Severe pneumonia, n (%) | Yes | 3 (10.0) | 0 | 0.54 |
|  | No | 27 (90.0) | 15 (100.0) |  |
| Multiple injuries, n (%) | Yes | 2(6.7) | 0 | 0.546 |
|  | No | 28 (93.3) | 15 (100.0) |  |
| Pancreatitis, n (%) | Yes | 4 (13.3) | 0 | 0.285 |
|  | No | 26 (86.7) | 15 (100.0) |  |
