# Supplementary material for: Identification of featured necroptosis-related genes and imbalanced immune infiltration in sepsis via machine learning
Source: Front Genet. 2023 Apr 6;14:1158029. doi: 10.3389/fgene.2023.1158029 (PMC10117955; doi:10.3389/fgene.2023.1158029)
Supplement: Supplementary file 4 [file Table3.DOCX]

**Supplementary Table 3:** qRT-PCR primer sequences of hub genes.

| **Genes** | **Forward primers** | **Reverse primers** |
| --- | --- | --- |
| *GATA3* | *5’-GCATCCAGACCAGAAACCGA-3’* | *5’-ACGAGCTGTTCTTGGGGAAG-3’* |
| *LEF1* | *5’-CCCGTGAAGAGCAGGCTAAA-3’* | *5’-AGGCAGCTGTCATTCTTGGA-3’* |
| *BCL2* | *5’-CCTTCTTTGAGTTCGGTGG-3’* | *5’-AGAAATCAAACAGAGGCCG-3’* |
| *BACH2* | *5’-CCAGCAACACCTCCGAGAAT-3’* | *5’-TCTTTCCTGGGCTGTTCGTC-3’* |
| *β-Actin* | *5’-CATGTACGTTGCTATCCAGGC-3’* | *5’-CTCCTTAATGTCACGCACGAT-3’* |
