# Supplementary material for: Identification of featured necroptosis-related genes and imbalanced immune infiltration in sepsis via machine learning
Source: Front Genet. 2023 Apr 6;14:1158029. doi: 10.3389/fgene.2023.1158029 (PMC10117955; doi:10.3389/fgene.2023.1158029)
Supplement: Supplementary file 5 [file Table4.DOCX]

**Supplementary Table 4:** The DE-NRGs of GSE65682.

| gene | conMean | treatMean | logFC | pValue | fdr |
| --- | --- | --- | --- | --- | --- |
| LEF1 | 6.036619 | 4.064692 | -0.57059 | 7.09E-26 | 1.25E-24 |
| BACH2 | 5.083627 | 3.613597 | -0.49242 | 8.4E-24 | 6.36E-23 |
| GATA3 | 5.214422 | 3.883701 | -0.42508 | 8.53E-27 | 4.52E-25 |
| MYC | 8.237372 | 6.366348 | -0.37172 | 1.58E-24 | 1.4E-23 |
| DNMT1 | 7.598691 | 6.012572 | -0.33777 | 2.35E-26 | 6.22E-25 |
| BCL2 | 4.675755 | 3.838283 | -0.28474 | 1.17E-24 | 1.24E-23 |
| FASLG | 3.802687 | 3.25768 | -0.22317 | 4.62E-20 | 2.72E-19 |
| HDAC9 | 4.345414 | 3.755639 | -0.21044 | 6.06E-13 | 2.01E-12 |
| CD40 | 4.391677 | 3.856773 | -0.18738 | 8.64E-14 | 3.05E-13 |
| SIRT1 | 6.035487 | 5.376157 | -0.16689 | 8.49E-14 | 3.05E-13 |
| TARDBP | 6.994635 | 6.232063 | -0.16654 | 4.58E-25 | 6.06E-24 |
| IDH2 | 4.975742 | 4.625462 | -0.10531 | 2.8E-09 | 5.94E-09 |
| BNIP3 | 4.873334 | 4.536422 | -0.10335 | 1.4E-06 | 2.4E-06 |
| CYLD | 6.132309 | 5.712366 | -0.10234 | 2.34E-14 | 9.53E-14 |
| DDX58 | 5.854944 | 5.495048 | -0.09152 | 0.000108 | 0.000147 |
| ITPK1 | 7.048792 | 6.651283 | -0.08374 | 1.15E-06 | 2.03E-06 |
| MAPK8 | 4.080927 | 3.869109 | -0.0769 | 5.78E-06 | 9.01E-06 |
| SQSTM1 | 7.522885 | 7.132582 | -0.07686 | 9E-10 | 2.07E-09 |
| MAP3K7 | 5.868587 | 5.611252 | -0.06469 | 4.24E-06 | 6.81E-06 |
| TNFRSF1B | 8.153128 | 7.805469 | -0.06287 | 3.86E-06 | 6.39E-06 |
| HSPA4 | 5.278599 | 5.055973 | -0.06217 | 5.02E-08 | 9.86E-08 |
| DIABLO | 6.268674 | 6.006718 | -0.06158 | 1.36E-08 | 2.77E-08 |
| USP22 | 6.460497 | 6.220769 | -0.05455 | 1.68E-09 | 3.72E-09 |
| CASP8 | 5.330481 | 5.139628 | -0.0526 | 9.55E-05 | 0.000133 |
| TSC1 | 5.50918 | 5.324806 | -0.04911 | 4.17E-07 | 7.9E-07 |
| CDKN2A | 3.494115 | 3.431275 | -0.02618 | 0.006935 | 0.008353 |
| HSP90AA1 | 7.271989 | 7.155483 | -0.0233 | 0.005657 | 0.006973 |
| ZBP1 | 4.573956 | 4.543719 | -0.00957 | 0.012631 | 0.014243 |
| SPATA2 | 4.090772 | 4.16402 | 0.025604 | 0.042415 | 0.046833 |
| TNFSF10 | 9.243849 | 9.410776 | 0.02582 | 0.009564 | 0.011019 |
| TRIM11 | 4.529105 | 4.666234 | 0.043033 | 0.001496 | 0.001887 |
| RIPK1 | 4.708191 | 4.858001 | 0.04519 | 0.00057 | 0.000755 |
| SLC39A7 | 4.008047 | 4.140896 | 0.047044 | 9.86E-06 | 1.49E-05 |
| SIRT2 | 4.319375 | 4.504274 | 0.060472 | 8.34E-07 | 1.52E-06 |
| BCL2L11 | 4.47968 | 4.677336 | 0.062291 | 2.13E-05 | 3.14E-05 |
| CFLAR | 8.089088 | 8.451159 | 0.063172 | 5.24E-10 | 1.26E-09 |
| APP | 4.599801 | 4.836333 | 0.072342 | 0.000894 | 0.001156 |
| FAS | 6.036459 | 6.350461 | 0.073159 | 0.007643 | 0.009001 |
| TNFRSF1A | 6.433115 | 6.854214 | 0.091474 | 8.53E-11 | 2.26E-10 |
| MLKL | 7.484281 | 8.020607 | 0.099848 | 9.62E-13 | 3E-12 |
| FADD | 6.26132 | 6.8062 | 0.120383 | 1.18E-16 | 5.23E-16 |
| KLF9 | 5.237297 | 5.728823 | 0.129416 | 5.45E-05 | 0.000078 |
| BRAF | 3.802995 | 4.200744 | 0.143509 | 1.45E-10 | 3.66E-10 |
| IDH1 | 5.464662 | 6.065078 | 0.150394 | 4.78E-11 | 1.33E-10 |
| RIPK3 | 4.168627 | 4.653685 | 0.158801 | 1.35E-20 | 8.92E-20 |
| ID1 | 3.381749 | 3.777217 | 0.159554 | 3.52E-19 | 1.87E-18 |
| FLT3 | 3.601386 | 4.317769 | 0.261734 | 2.08E-12 | 6.14E-12 |
| HAT1 | 4.564996 | 5.725938 | 0.326898 | 2.15E-17 | 1.04E-16 |
