# Supplementary material for: Identification of featured necroptosis-related genes and imbalanced immune infiltration in sepsis via machine learning
Source: Front Genet. 2023 Apr 6;14:1158029. doi: 10.3389/fgene.2023.1158029 (PMC10117955; doi:10.3389/fgene.2023.1158029)
Supplement: Supplementary file 6 [file Table6.DOCX]

**Supplementary Table 6:** Immune cells proportion of blood routine between septic patients and healthy controls.

| **Characteristics** |  | **Septic patients** | **Healthy controls** | **p-Value** |
| --- | --- | --- | --- | --- |
|  |  | **(n=30)** | **(n=15)** |  |
| Lymphocytes (%) | Mean | 7.0 | 26.7 | ＜0.001 |
|  | SD | 4.1 | 10.9 |  |
| Monocytes (%) | Mean | 5.5 | 6.9 | 0.193 |
|  | SD | 4.0 | 1.5 |  |
| Neutrophils (%) | Mean | 86.0 | 63.4 | ＜0.001 |
|  | SD | 7.7 | 11.6 |  |
| Eosinophils (%) | Mean | 1.2 | 2.6 | 0.053 |
|  | SD | 1.9 | 2.8 |  |
| Basophils (%) | Mean | 0.2 | 0.5 | ＜0.001 |
|  | SD | 0.2 | 0.3 |  |
